# 2-Hydroxypropyl-β-Cyclodextrin-Based Complexes Improve Polyphenol Solubility and Bioaccessibility: Evaluation by Validated HPLC–DAD Method

**DOI:** 10.3390/molecules31040600

**Published:** 2026-02-09

**Authors:** Eleonora Perak Junaković, Anja Vujnović, Nada Oršolić, Svjetlana Terzić, Miroslav Andrišić, Miroslav Benić, Dominika Fajdić, Sonja Sinković, Katja Vretenar Špigelski, Irena Žarković, Ksenija Šandor

**Affiliations:** 1 Laboratory for Analysis of Veterinary-Medicinal Products, Croatian Veterinary Institute, Savska cesta 143, 10000 Zagreb, Croatia; terzic.svjetlana@gmail.com (S.T.); andrisic@veinst.hr (M.A.); fajdic@veinst.hr (D.F.); sinkovic@veinst.hr (S.S.); zarkovic@veinst.hr (I.Ž.); sandor@veinst.hr (K.Š.); 2 Division of Animal Physiology, Faculty of Science, University of Zagreb, Rooseveltov trg 6, 10000 Zagreb, Croatia; nada.orsolic@biol.pmf.hr; 3 Laboratory for Mastitis and Raw Milk Quality, Croatian Veterinary Institute, Savska cesta 143, 10000 Zagreb, Croatia; benic@veinst.hr; 4 Regulatory and Pharmacovigilance Department, Oktal Pharma d.o.o., Utinjska Ulica 40, 10000 Zagreb, Croatia; katja.vretenarspigelski@oktal-pharma.hr

**Keywords:** propolis extracts, polyphenols, cyclodextrins, lyophilization, HPLC–DAD, method validation, static in vitro digestion, bioaccessibility

## Abstract

Propolis is a rich natural source of biologically active polyphenols; however, their therapeutic potential is often limited by poor oral bioaccessibility and bioavailability. This study reports the development and validation of a high-performance liquid chromatography–diode array detector (HPLC–DAD) method optimized for the quantification of major propolis polyphenols—caffeic acid (CA), pinocembrin (PC), chrysin (CR), caffeic acid phenethyl ester (CAPE), and galangin (GN) in 2-hydroxypropyl-β-cyclodextrin (HP-β-CD)-based complexes. A green complexation approach based on HP-β-CD and lyophilization was applied to continental propolis, yielding a water-soluble formulation suitable for oral administration. The isocratic HPLC–DAD method demonstrated linearity, sensitivity, and precision, suitable for reliable analysis of polyphneols in cyclodextrin-based matrices. Gastrointestinal behavior was evaluated using a simulated oral, gastric, and intestinal digestion model. PC and CAPE remained stable throughout digestion, whereas GN, CR, and CA showed higher sensitivity, with decreases of 43.1–71.6% compared to undigested samples. HP-β-CD complexation enhanced polyphenol solubility and improved gastrointestinal stability. Intestinal bioaccessibility, assessed by a centrifugation model, ranged from 77.2% (CR) to 124.9% (CA). However, the complexes did not permeate the artificial intestinal membrane, resulting in reduced dialyzable polyphenols, with CA being undetectable. These findings provide a validated analytical platform and mechanistic insight into the gastrointestinal behavior of cyclodextrin-complexed propolis polyphenols, supporting their application in oral functional formulations.

## 1. Introduction

Propolis is a resinous material produced by honeybees from plant exudates and used to seal and protect the hive. In recent decades, propolis and its extracts have attracted substantial interest in pharmaceutical and biomedical research due to their broad biological activities, including antimicrobial, antioxidant, anti-inflammatory, immunomodulatory, and anticancer effects [1,2,3]. These effects are primarily attributed to propolis polyphenols, particularly flavonoids and phenolic acids, which represent chemically diverse, bioactive constituents of growing relevance for pharmaceutical, nutraceutical, and functional food applications [4,5].

A major analytical challenge associated with propolis-based products is the pronounced variability of their chemical composition, which depends strongly on botanical source and geographical origin, thereby complicating standardization, comparability, and quantitative assessment [4,6]. Poplar-type propolis, common in temperate regions of Europe, exhibits a relatively consistent profile dominated by flavonoid aglycones such as pinocembrin (PC), chrysin (CR), and galangin (GN), together with phenolic acids and esters including caffeic acid (CA) derivatives and caffeic acid phenethyl ester (CAPE). These compounds are therefore frequently selected as marker analytes for quality control, method development, and standardization studies [7,8]. In addition, extraction technology and solvent selection significantly influence extract composition and analytical behavior. Recent studies have demonstrated that alternative extraction systems can yield chemically comparable propolis extracts while altering matrix complexity and usability relative to traditional ethanolic extracts [9,10].

Despite extensive bioactivity data, quantitative evaluation of propolis polyphenols remains challenging due to poor aqueous solubility, limited chemical stability, and susceptibility to degradation under gastrointestinal conditions, which constrain systemic exposure and complicate quantification in digestion matrices [11,12,13,14,15]. To address solubility- and stability-related limitations, various delivery strategies have been investigated, including cyclodextrins, nanoparticles, and liposomes [16,17,18,19]. From an analytical perspective, cyclodextrin-based systems are particularly relevant because they form non-covalent host–guest complexes with polyphenols and can modify apparent solubility and stability without chemically altering the analytes [20,21]. 2-hydroxypropyl-β-cyclodextrin (HP-β-CD) is widely used for flavonoids and phenolic esters and is generally regarded as a solubilizer and stabilizer rather than a direct permeation enhancer. Thus, any enhancement in uptake is expected to be indirect and dependent on dissolution and stability as limiting factors [22,23]. Recent studies have shown that cyclodextrin complexation can preserve polyphenol integrity and increase the measurable bioaccessible fraction during simulated gastrointestinal digestion, including propolis preparations [24,25,26].

In contrast, delivery systems based on nanoparticles and liposomes rely on encapsulation within carrier matrices or phospholipid bilayers and can further protect labile compounds but often introduce additional analytical complexity (e.g., matrix interferences and variable release kinetics) and may require more elaborate manufacturing control [27,28,29]. For example, superparamagnetic nanocarriers have been shown to modulate the release kinetics of poorly soluble flavonoids under external magnetic fields, highlighting how morphology of magnetic nanoparticles and polymer coating can protect polyphenols from poor solubility, premature degradation, and low bioavailability [28]. Liposomes, which incorporate polyphenols into phospholipid bilayers, can facilitate incorporation into bile salt–mixed micelles during digestion and potentially enhance both bioaccessibility and uptake. However, their application may be constrained by physicochemical stability and treatment challenges. Processing strategies such as spray-drying and freeze-drying (lyophilization) are frequently combined with such systems to improve stability and handling, although they can also alter extract composition and extraction efficiency, reinforcing the need for validated analytical methods [26,30].

Accurate assessment of polyphenol stability, release, and bioaccessibility, therefore, requires sensitive and selective analytical methods capable of simultaneously quantifying structurally related compounds in complex digestion matrices. High-performance liquid chromatography coupled with diode array detection (HPLC–DAD) remains a robust and widely used technique for polyphenol analysis and quality control [8,31]. Recent method development emphasizes comprehensive validation, system suitability, and performance-based approaches for analytical procedures [32,33,34]. Moreover, harmonized static in vitro digestion models, such as INFOGEST [35], complement these advances by ensuring reproducible digestion conditions and enabling meaningful comparison of analytical outcomes across studies.

Although propolis delivery systems are increasingly studied, fully validated analytical data on the gastrointestinal fate of key poplar-type polyphenols remain limited, especially for cyclodextrin-based complexes. Therefore, the present study aimed to: (i) develop and validate a robust HPLC–DAD method for the simultaneous quantification of representative poplar-type propolis polyphenols; (ii) apply this method to monitor polyphenol stability and release during simulated oral, gastric, and intestinal digestion phases; and (iii) quantify the bioaccessible and dialyzable fractions delivered from a lyophilized propolis–HP-β-CD complex. By emphasizing analytical reliability alongside physiologically relevant digestion conditions, this work provides a methodological framework for the standardized evaluation of propolis-based formulations.

## 2. Results

### 2.1. Development and Optimization of HPLC–DAD Method

Method development was performed using a standard mixture of the five target polyphenols: CA, PC, CR, CAPE, and GN. Chromatographic optimization focused on achieving complete baseline separation with acceptable peak symmetry, retention behavior, and analysis time.

Reversed-phase C-18 columns were selected due to their robustness, pH stability, and established suitability for polyphenol analysis. Initial experiments employing longer columns (250 mm) and gradient elution resulted in suboptimal resolution, broader peak shapes, and increased variability and were therefore excluded from further validation. Improved separation efficiency was achieved using shorter C-18 columns with smaller particle size, which provided sharper peaks and enhanced reproducibility.

Mobile-phase composition was optimized using water acidified with formic acid as the aqueous component and methanol as the organic modifier. Isocratic elution was selected over gradient elution, as it yielded more consistent retention times, improved peak symmetry, and reduced run-to-run variability. Chromatographic separation was therefore performed using solvent A (water/methanol/formic acid, 97:2:1, *v*/*v*/*v*) and solvent B (methanol) at a 45:55 (*v*/*v*) ratio (A:B).

Under the optimized conditions (Table 1), all five analytes were fully separated within 35 min, with a total run time of 40 min.

System suitability parameters confirmed the robustness of the method (Table 2). Retention factors ranged from 0.5 to 17.4, asymmetry factors were ≤1.6 for all compounds, and theoretical plate numbers exceeded 9000. Resolution values between adjacent peaks (Rs = 3.1–50.9) were well above the acceptance criterion (Rs > 2), confirming baseline separation. Low variability in retention times and peak areas across replicate injections further supported method reliability.

A representative chromatogram recorded at 290 nm under optimized conditions is shown in Figure S1. Although isocratic operation required periodic column washing and precolumn replacement to maintain performance, the method provided reproducible separation, adequate sensitivity, and robust chromatographic performance suitable for subsequent validation and application in digestion studies.

### 2.2. Method Validation

#### 2.2.1. Selectivity

Selectivity was assessed by comparing chromatograms of solvent blanks, Dubrovnik (Dub) propolis-HP-β-CD-based complex without target analytes, a spiked Dub sample, and HP-β-CD complexes with propolis from Valpovo (Vlp) and Zagreb (Zg). No detectable peaks co-eluted with the target analytes at their respective retention times. Signal-to-noise ratios (S/N) at the LOD and LOQ levels met commonly accepted criteria, confirming that the method was selective for CA, PC, CR, CAPE, and GN in complex propolis matrices.

#### 2.2.2. Linearity and Sensitivity

High linearity was obtained for all analytes across the examined concentration ranges (Table 3), with correlation coefficients (R^2^) ≥ 0.9958. The residuals were randomly distributed across the calibration range, indicating no systematic deviation from linearity. Limits of detection (LOD) and quantification (LOQ) were in the range 0.10–1.84 μg mL^−1^ and 0.32–5.58 μg mL^−1^, respectively, confirming adequate sensitivity for the expected analyte levels in propolis samples.

#### 2.2.3. Precision

Repeatability (intra-day precision) and intermediate precision (inter-day precision) were evaluated using replicate injections of standard solutions and propolis complexes. For standard solutions, the relative standard deviation (% RSD) of peak areas remained below 2%, indicating good repeatability. As shown in Table S1, the % RSD values for the propolis complexes did not exceed 8.5%, indicating acceptable precision for complex biological matrices. The relatively low interday % RSD values indicate that the method is precise and provides reproducible quantitative results under routine analytical conditions.

#### 2.2.4. Accuracy

Accuracy was evaluated through recovery experiments in a Dub propolis complex spiked at three concentration levels (LOQ, nominal, and 150% of nominal concentration). Mean recoveries ranged from 80.0% to 110.0% for all analytes (Table 4), with associated % RSD values generally below 5%. Due to the complexity of natural matrices, slightly broader recovery ranges are commonly accepted in method validation protocols. These findings indicate that the method is both accurate and precise over the tested range and meets commonly accepted criteria for quantitative analytical methods.

#### 2.2.5. Robustness and Stability

Robustness was tested by making minor modifications to chromatographic parameters. Reducing the formic acid content and the proportion of component A in the mobile phase did not significantly affect resolution, peak area, or theoretical plate count, although it shortened the analysis time. In contrast, increasing the formic acid content prolonged analysis and reduced peak responses, notably impairing the separation of CR and CAPE (Figure S2). Temperature variations (±3 °C) affected retention time without compromising resolution. Increasing the injection volume from 5 to 10 µL proportionally increased the peak area, with no negative impact on chromatographic performance.

Further robustness assessment using different C18 column batches yielded consistent results. Stability studies confirmed that prepared standard and sample solutions remained stable for up to 72 h at room temperature, 60 days under refrigerated conditions (2–8 °C), and through two freeze–thaw cycles over 14 days. In all cases, % RSD values remained below 2%, confirming method robustness and solution stability under routine laboratory conditions.

### 2.3. Application to Propolis-HP-β-CD Complexes

The validated RP–HPLC–DAD method was applied to aqueous suspensions of lyophilized preparations obtained from 70% EEP and HP-β-CD, using continental propolis samples from Vlp and Zg. In these samples, HP-β-CD was present in the final aqueous preparation at a concentration of 20 mg mL^−1^. For comparison, an aqueous preparation without HP-β-CD was produced using the same procedure, including solvent evaporation and lyophilization of the ethanolic propolis extract, followed by reconstitution in water. This ensured that both the HP-β-CD-containing and aqueous (control) preparations were in a comparable physical state before analysis and differed only with respect to the presence of the cyclodextrin carrier. The influence of HP-β-CD on the apparent solubility of polyphenols is presented in Table 5 and Figure S3.

**Figure 1:**
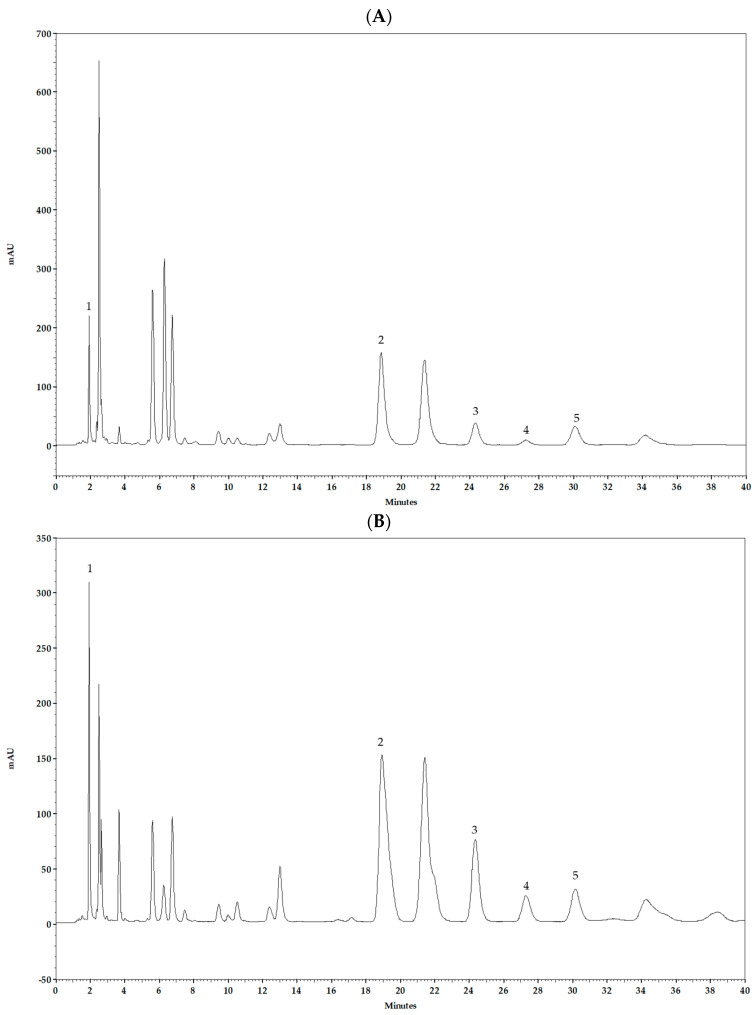
Chromatograms showing identified polyphenols caffeic acid (CA), pinocembrin (PC), chrysin (CR), caffeic acid phenethyl ether (CAPE), and galangin (GN) in the HP-β-CD-based complex with propolis from Valpovo (**A**), and from Zagreb (**B**) at 290 nm, obtained under optimal conditions of the method (1: CA, 2: PC, 3: CR, 4: CAPE, 5: GN).

The lyophilized product prepared without HP-β-CD resulted in resinous, poorly soluble residue. In contrast, the preparation obtained with HP-β-CD yielded homogeneous, fully lyophilized powder, indicating improved formulation characteristics. Polyphenols in HP-β-CD-based complexes and aqueous preparation were identified by comparing the retention times of the eluting peaks with those of the standards. Quantitative analysis showed that HP-β-CD complexation significantly increased the solubility of less polar compounds PC, CR, GN, and CAPE compared with aqueous preparation without HP-β-CD (Table 5). For these compounds, the degree of increased solubility ranged from approximately six-fold (PC) to eighteen-fold (CR). Conversely, CA exhibited substantially higher concentrations in the aqueous preparation without HP-β-CD (5224.7 ± 17.5 μg mL^−1^) than in the HP-β-CD complex (248.1 ± 3.9 μg mL^−1^). Under the validated HPLC conditions, HP-β-CD-based complexes from two continental propolis samples showed a similar polyphenolic profile (Figure 1A,B).

### 2.4. Analysis of Fractions from In Vitro Digestion

**Figure 2:**
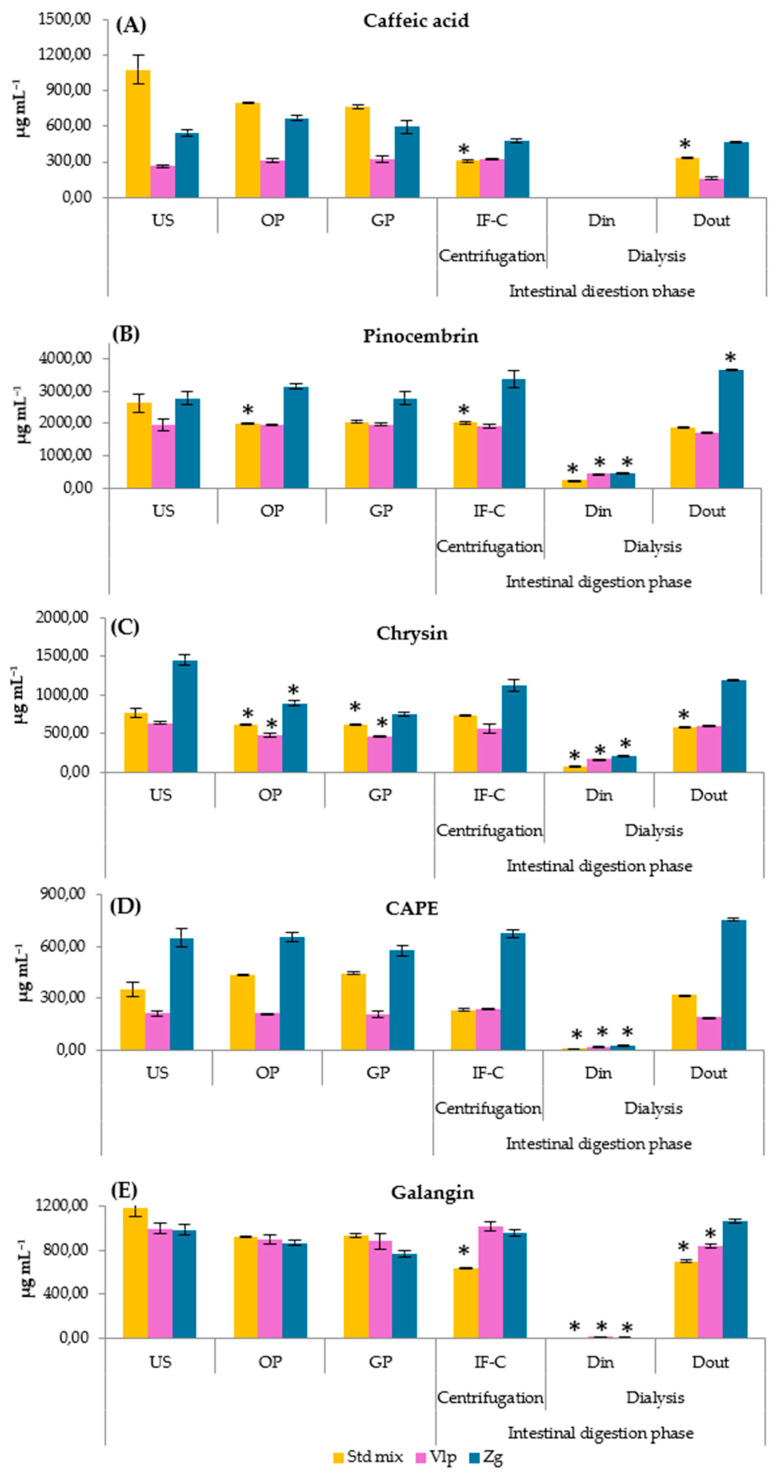
(**A**–**E**) Concentration changes of polyphenols: (**A**) caffeic acid, (**B**) pinocembrin, (**C**) chrysin, (**D**) caffeic acid phenethyl ether, (**E**) galangin, during in vitro digestion phases in HP-β-CD-based complexes of standard mixture (Std mix), Valpovo (Vlp) and Zagreb (Zg) propolis samples. Results are expressed as mean mass concentration ± standard deviation (SD) (*n* = 3). US, undigested sample; OP, oral phase; GP, gastric phase; IF-C, soluble small-intestinal fraction obtained after centrifugation; Din and Dout, fractions inside and outside the semipermeable membrane, respectively. Statistical significance was assessed using Dunn’s post hoc test (*p* < 0.05); * indicates a significant difference compared with the undigested sample (US) for the same preparation.

Quantitative analysis revealed pronounced, compound-specific differences in the gastrointestinal stability of the investigated polyphenols (Figure 2A–E).

As presented in Figure 2B, PC showed the highest stability across all formulations. In the standard mixture, PC concentrations decreased modestly from 2634.7 µg mL^−1^ (US) to 2006.5 µg mL^−1^ after the oral phase and remained stable during gastric digestion, and the intestinal soluble concentration in IF-C was 2023.0 µg mL^−1^, which was comparable to undigested levels. In propolis complexes, PC was even more stable, increasing in the Zg sample from 2769.7 µg mL^−1^ (US) to 3356.7 µg mL^−1^ in IF-C.

CAPE similarly exhibited high digestive stability (Figure 2D). In the standard mixture, CAPE increased from 349.9 µg mL^−1^ (US) to 446.4 µg mL^−1^ after gastric digestion, with partial redistribution in the intestinal phase. In propolis complexes, CAPE concentrations were well preserved, increasing slightly in the Zg sample from 648.9 µg mL^−1^ (US) to 674.5 µg mL^−1^ in IF-C and reaching 756.6 µg mL^−1^ in the Dout fraction.

Figure 2C shows that CR exhibited intermediate stability. In the standard mixture, CR decreased from 761.9 µg mL^−1^ to 610.8 µg mL^−1^ after the oral phase but partially recovered in the IF-C to 737.6 µg mL^−1^. In propolis complexes, CR concentrations in IF-C remained high, particularly in the Zg sample (1113.7 µg mL^−1^ vs. 1447.1 µg mL^−1^ in US). In contrast, CA and GNwere the most digestion-sensitive polyphenols, as can be observed in Figure 2A,E. GN concentrations in the standard mixture decreased from 1177.7 µg mL^−1^ to 637.8 µg mL^−1^ in IF-C, with similar losses observed in propolis complexes. CA showed the greatest variability; in the standard mixture, CA declined sharply from 1078.7 µg mL^−1^ (US) to 305.7 µg mL^−1^ in IF-C, whereas in the Vlp complex, CA increased from 264.3 µg mL^−1^ to 363.9 µg mL^−1^ in IF-C. Overall, based on concentration retention relative to undigested samples, gastrointestinal stability followed the order: PC ≈ CAPE > CR > GN ≈ CA.

This ranking was consistent across the standard mixture and both propolis complexes, with matrix-dependent effects influencing the magnitude but not the direction of stability trends.

### 2.5. Bioaccessibility of Individual Polyphenols

Bioaccessibility of individual polyphenols was evaluated using two complementary in vitro digestion approaches. In the centrifugation-based model, bioaccessibility was defined as the soluble intestinal fraction (IF-C). In the dialysis-based model, the soluble intestinal fraction was further partitioned into dialyzable (Din) and non-dialyzable (Dout) fractions. Overall bioaccessibility was expressed as the proportion of compounds recovered in the intestinal supernatant after centrifugation, calculated as (Din + Dout)/US. This approach also distinguished the potentially absorbable fraction (Din) from the soluble but non-permeating fraction (Dout), representing compounds that remained in solution yet did not cross the dialysis membrane.

Table 6 reports the bioaccessibility and dialyzability (%) values for individual phenolic compounds in three HP-β-CD-based complexes after simulated gastrointestinal digestion. For the standard polyphenol mixture, bioaccessibility values obtained using the centrifugation model ranged from 28.5% for CA to 97.2% for CR. When expressed as the total soluble fraction in the dialysis model (Din + Dout)/US, bioaccessibility ranged from 31.5% (CA) to 94.2% (CAPE). Dialyzable fractions were consistently low, reaching only 0.8% for GN and 2.6% for CAPE, while CA showed no detectable dialyzable fraction, indicating limited permeability across the dialysis membrane and partial losses during digestion.

Encapsulation within the Vlp propolis complex markedly enhanced bioaccessibility in both models. Using the centrifugation-based approach, IF-C/US values increased to 98.2% for PC, 102.3% for GN, and 112.0% for CAPE, while CA exhibited the largest relative improvement, increasing more than fourfold compared with the std mix. In the dialysis-based model, total bioaccessibility reached 111.1% for PC and 121.8% for CR. Dialyzability also increased, particularly for PC (22.9%) and CR (26.5%), whereas GN (2.1%) and CAPE (8.8 ± 1.1%) remained weakly dialyzable.

The Zg propolis complex exhibited the most pronounced effects overall. PC showed the highest bioaccessibility, with IF-C/US values of 121.6% and 148.8% in the dialysis model. CAPE and GN also displayed high intestinal solubilization, reaching 104.2% and 97.4%, respectively, with corresponding total bioaccessibility values exceeding 100%. In contrast, CR exhibited lower intestinal solubilization (IF-C/US 77.2%) and limited dialyzability (14.8%), while CA again showed no detectable dialyzable fraction despite substantial bioaccessibility (88.2%).

Across all formulations, dialyzable fractions remained low relative to total bioaccessibility and were consistently measurable only for PC and CR, while GN, CA, and CAPE were largely retained within the non-dialyzable fraction. These results indicate that although encapsulation significantly enhanced intestinal solubilization, only a limited proportion of polyphenols was capable of crossing the dialysis membrane, as further illustrated by the chromatographic profiles shown in Figure S4.

## 3. Discussion

### 3.1. Study Design and Interpretation Framework

This study provides a compound-resolved evaluation of gastrointestinal stability, intestinal availability, and potential permeability of major poplar-type propolis polyphenols encapsulated in HP-β-cyclodextrin (HP-β-CD) using the standardized three-phase INFOGEST in vitro digestion model [35]. Inclusion of the oral phase, which is often omitted in earlier digestion studies, improves physiological relevance and ensures alignment with internationally accepted protocols, strengthening comparability and translational relevance [36,37,38]. A key prerequisite for interpreting digestion-driven changes in such chemically complex systems is analytical reliability. Therefore, all target polyphenols (CA, PC, CR, CAPE, GN) were quantified using a purpose-developed RP-HPLC-DAD method that was optimized to achieve complete baseline separation and subsequently validated in accordance with ICH Q2(R2) and Q14 guidelines [33,34]. This aspect is particularly relevant in the present context because both propolis and digestion samples represent analytically demanding matrices. They contain co-extracted compounds, enzymatic digestion products, bile components, and HP-β-CD-associated species that may compromise chromatographic specificity. Collectively, the performance characteristics substantially strengthen confidence that observed differences between undigested samples and intestinal fractions (IF-C, Din, Dout), including low dialyzable levels and occasional apparent over-recoveries, reflect true digestion- and formulation-driven changes.

Because interpretation of polyphenol fate depends on how intestinal bioaccessibility is defined, two complementary approaches were applied under identical digestion conditions: centrifugation of intestinal chyme to determine bioaccessibility in soluble intestinal fraction and dialysis through a semipermeable membrane to estimate dialyzability. In line with current consensus, these parameters were treated as sequential but conceptually distinct, as neither alone equates to true bioavailability, which additionally depends on epithelial transport, metabolic transformation, and systemic distribution [11,12]. Thus, the dialysis model provides mechanistic insight into gastrointestinal release and potential passive permeability but does not enable direct quantification of absolute bioavailability [15,39,40]. Within this framework, the dialyzable fraction (Din) should be interpreted as an indicator of potential passive permeability rather than direct intestinal absorption in vivo [41,42,43,44]. Overall, the findings highlight that the choice of intestinal assessment model substantially shapes conclusions, reinforcing the need to clearly distinguish bioaccessibility, dialyzability, and bioavailability and to apply complementary approaches in parallel [45,46].

### 3.2. Solubility and Compound-Specific Behavior in HP-β-CD Systems

Against this methodological background, the formulation effect of HP-β-CD became evident already at the level of apparent aqueous solubility. Complexation with HP-β-CD markedly increased the apparent aqueous solubility of hydrophobic flavonoids and CAPE, thereby enlarging the fraction available for intestinal interaction. This is consistent with the established role of cyclodextrins as pharmaceutical solubilizers and stabilizers for poorly water-soluble polyphenols [18,20,47,48] and is supported by studies on phenolic–HP-β-CD-based systems [49,50,51]. In contrast, CA showed higher apparent solubility in aqueous preparation without HP-β-CD, reflecting its polarity and ionizable carboxyl group that promote hydration and partial dissociation in aqueous media [52]. Due to its limited hydrophobic surface, CA is less favorably accommodated within the hydrophobic cavity of HP-β-CD and likely interacts weakly at the cyclodextrin rim, reducing the proportion of freely dissolved CA in undigested complex systems [17,50,51,52]. These observations confirm that cyclodextrin-mediated solubilization is compound-specific and governed by molecular size, polarity, and ionization behavior [21,23]. The observed enhancement reflects apparent encapsulation performance rather than true encapsulation efficiency, as free and complexed polyphenols were not experimentally separated [18,20]. Therefore, the obtained products are best described as HP-β-CD-based complex systems rather than structurally confirmed inclusion complexes.

### 3.3. Encapsulation Effects on Gastrointestinal Stability and Bioaccessibility

Importantly, the improved apparent solubility translated into clearer differences during simulated digestion, particularly in the intestinal phase, where bioaccessibility is operationally defined. Standard mixture complexes were prepared to enable direct comparison of polyphenol digestion behavior in the absence of the propolis matrix and to isolate the contribution of HP-β-CD complexation from matrix-derived effects. Encapsulation of propolis extracts in HP-β-CD improved gastrointestinal stability and increased bioaccessibility compared with the standard mixture complex. As summarized in Table 6, intestinal soluble fractions (IF-C) and corresponding bioaccessibility values increased particularly for PC, GN, and CAPE in both Vlp and Zg complexes, with IF-C/NU ratios shifting from below unity in the standard mixture to values approaching or exceeding 100% after encapsulation of extracts. Similar improvements in polyphenol bioaccessibility after cyclodextrin encapsulation have been reported for olive pomace and other phenolic-rich matrices [46,51,53]. The strongest gains were observed for PC and CAPE, whose intestinal bioaccessibility increased from ~70–80% to ~100–120% in HP-β-CD-based complexes, while GN increased from ~50–55% to ~95–100%. For CA, HP-β-CD produced a matrix-dependent response, with markedly increased bioaccessibility in the Vlp complex (~125%) and more moderate stabilization in the Zg complex (~85–90%).

### 3.4. Explaining Recoveries Above 100% Under INFOGEST Conditions

In this context, bioaccessibility values above 100%, most notably for CA in the Vlp complex, are best interpreted as an INFOGEST-related operational phenomenon rather than as true mass-balance recovery. Such over-recoveries are widely reported in digestion studies of phenolic-rich matrices and are generally attributed to digestion-induced liberation and enhanced solubilization of compounds that are partially inaccessible or poorly extractable in undigested samples, rather than to analytical artefacts [41,42,45,51,54]. Mechanistically, pH transitions between gastric and intestinal phases, together with pancreatin activity and bile salts inherent to the INFOGEST protocol, can alter host–guest equilibria of HP-β-CD and promote partial dissociation of cyclodextrin–polyphenol inclusion structures, increasing soluble and detectable guest concentrations in the intestinal supernatant [25,49,50,51]. Enzymatic digestion and matrix disintegration may further liberate matrix-bound phenolics not fully extractable prior to digestion, elevating apparent recoveries [46,54,55]. Because bioaccessibility in the centrifugation-based INFOGEST model is operationally defined as the soluble intestinal fraction, this fraction may also include bile-salt micelles and fine colloidal material that increase apparent recoveries [54,56]. Collectively, these effects reflect improved extractability and solubilization under simulated gastrointestinal conditions rather than true mass-balance recovery or de novo formation.

### 3.5. Phase- and Structure-Dependent Digestive Stability Patterns

Beyond these operational considerations, the data also reveal clear structure-driven differences in digestive stability. Across all formulations, polyphenols changed minimally during oral and gastric digestion, whereas the intestinal phase was decisive for both apparent bioaccessibility and potential permeability. Marked compound-specific differences were observed: caffeic acid (CA) and galangin (GN) were the most digestion-sensitive constituents, with CA decreasing by ~12–72% and GN by ~3–46% in the intestinal soluble fraction, depending on matrix; losses were greatest in the standard mixture and partially mitigated by HP-β-CD complexation. In contrast, PC and CAPE displayed the highest gastrointestinal stability, with intestinal concentrations remaining close to or exceeding those in undigested samples, particularly in the Zagreb complex. Chrysin (CR) showed intermediate behavior. Overall, the stability ranking (PC ≈ CAPE > CR > GN ≈ CA) supports a structure-driven response in which aromatic conjugation and moderate hydrophobicity favor resistance to gastrointestinal conditions and stronger cyclodextrin interactions [20,21,48].

### 3.6. Divergence Between Intestinal Bioaccessibility and Dialyzability

While these patterns demonstrate improved luminal availability, they also clarify an important limitation—increased bioaccessibility does not necessarily imply increased permeability. Despite improved solubility and bioaccessibility, dialyzable fractions remained consistently low for most analytes, indicating that HP-β-CD complexation mainly increases luminal availability and stability rather than promoting passive permeation through the artificial membrane. Only PC and, to a lesser extent, CR showed measurable dialyzability, whereas CA, GN, and CAPE were largely retained in the non-dialyzable fraction. Similar observations for cyclodextrin-encapsulated polyphenols have been reported by Jug et al. [51], highlighting that dissociation is likely required prior to absorption and that artificial membrane models do not capture transporter-mediated uptake or dilution-driven dissociation dynamics. Accordingly, cell-based systems such as Caco-2 monolayers represent an important next step for validating intestinal uptake potential [19].

### 3.7. Matrix Effects and Source-Dependent Differences Between Vlp and Zg Complexes

In addition to compound structure and model choice, the propolis source itself appears to shape the magnitude of these effects. Differences between Vlp and Zg propolis complexes indicate that the initial propolis composition influences complexation behavior and digestive outcomes. Zg samples generally exhibited higher IF-C, Din, and Dout values for PC and CAPE (*p* < 0.05), consistent with source-dependent variation in the proportion of resinous (balsam) and wax fractions [4,6] and with compositional effects on polyphenol bioaccessibility discussed by Radić et al. [46]. Additionally, our previous study reported by Perak Junaković et al. [57] demonstrated significantly higher total phenolic content in Zagreb propolis relative to Valpovo propolis, suggesting a higher balsam-to-wax ratio and a matrix more favorable for cyclodextrin interaction and stabilization.

### 3.8. Implications for Formulation Development

Taken together, and considering both the formulation-driven solubility gains and the model-dependent interpretation of intestinal availability, these results demonstrate that HP-β-cyclodextrin complexation effectively improves apparent solubility, gastrointestinal stability, and bioaccessibility of selected propolis polyphenols, particularly PC and CAPE, while exerting only limited influence on passive permeability. This distinction emphasizes the need to differentiate solubility-driven bioaccessibility from true intestinal absorption when evaluating delivery systems for polyphenol-rich natural products [45,46]. Given the favorable safety profile, regulatory acceptance, and green chemistry credentials of HP-β-CD, the findings support its further development as a versatile platform for more standardized propolis-based nutraceutical and functional formulations [48,58].

## 4. Materials and Methods

### 4.1. Standards and Reagents

Phenolic compound standards used in this study include GN, CR, CA, andCAPE purchased from Sigma-Aldrich, Shanghai, China; PC from Sigma-Aldrich, Tashkent, Uzbekistan; and HP-β-CD with the molar substitution of 0.8 and average molecular weight of 1460 Da from Wacker Chemie AG, Burghausen, Germany. Chemicals sodium chloride (NaCl, ACS reag.), magnesium chloride hexahydrate (MgCl_2_·6H_2_O, Bioreag.), and ammonium carbonate ((NH_4_)_2_CO_3_ ACS reag.) were obtained from Sigma-Aldrich, Steinheim, Germany, while ethanol, absolute, was purchased from Supelco, Darmstadt, Germany.

For HPLC development and analysis, methanol, HPLC-grade, and acetonitrile were purchased from Honeywell (Seelze, Germany), while formic acid for LC-MS, ~98%, was from Honeywell (Mosbach, Germany), and HPLC-grade water was obtained from Honeywell (Darmstadt, Germany).

The reagents used in in vitro digestion protocol were α-amylase from human saliva (XIII-A type, 1099 units/mg), pepsin from porcine gastric mucosa (lyophilized powder; 4472 units/mg), pancreatin from porcine pancreas (digestion power: 8× United States Pharmacopoeia), and beef bile (dry, unfractionated), all purchased from Sigma-Aldrich, St. Louis, MO, USA, SAD. Cellulose dialysis membrane (molecular weight cutoff of 3.5 kDa) was obtained from Spectrum Labs (New Brunswick, NJ, USA). All other reagents and solvents used were of analytical grade.

### 4.2. Propolis Samples

Raw propolis samples were supplied from two bee farms in Croatia’s continental area (Valpovo and Zagreb) during autumn 2019 and 2020. The propolis sample from Dubrovnik (Dub sample), used in validation studies, was obtained from the southern Adriatic location. The samples were kept at −10 to −20 °C in sealed plastic bags until extraction.

### 4.3. Encapsulation of EEP with HP-β-CD

The HP-β-CD-based complex was prepared according to the protocol described by Perak Junaković et al. [25]. Briefly, frozen propolis was homogenized, and 0.5 g of powder was extracted with 5 mL of 70% ethanol by sonication (35 kHz, 25 °C, 1 h). After centrifugation (4472× *g*, 20 min, 4 °C), the extract was stored overnight at −10 to −20 °C and centrifuged again under identical conditions. The supernatant was filtered (Whatman No. 4), adjusted to 10 mL with 80% ethanol, and evaporated to dryness under reduced pressure at 50 °C to obtain the ethanolic extract of propolis (EEP). The dry extract was suspended in HP-β-cyclodextrin aqueous solution using an approximately 1:2 (*w*/*w*) extract:cyclodextrin ratio, and sonicated for 2 h (35 kHz, 25 °C). As commonly reported for multicomponent plant extracts, HP-β-CD was used in moderate excess to promote interaction with polyphenolic constituents. The suspension was concentrated under reduced pressure (72 mbar, 50 °C), frozen at −80 °C, and lyophilized (−50 °C, 0.1–0.3 mbar). Aqueous preparation was obtained using the identical procedure, differing only in the absence of cyclodextrin.

In addition, standard mixture complexes were prepared at the following concentrations of polyphenols: PC (40 μL·mL^−1^), CR (12 μL·mL^−1^), GN (20 μL·mL^−1^), CA (3.0 μL·mL^−1^), and CAPE (4.0 μL·mL^−1^). The lyophilized powders prepared from Valpovo and Zagreb propolis, aqueous preparation, and the standard mixture (CA, PC, CR, CAPE, and GN) were stored at −20 °C until analysis.

Encapsulation performance was evaluated indirectly based on solubility enhancement of individual polyphenols following complexation with HP-β-CD. Direct determination of encapsulation efficiency (i.e., separation of free and complexed compounds) was not performed.

### 4.4. HPLC Analysis

#### 4.4.1. Solution Preparation

Stock solutions (1.0 mg/mL) of the certified reference materials CA, PC, CR, CAPE, and GN were prepared in methanol and then diluted with the mobile phase before HPLC analysis. Calibrator solutions were made by appropriately diluting the stock solutions to produce mixtures of the standards. The concentration ranges for the individual polyphenols were as follows: CA 2–70 μg/mL, PC 2–70 μg/mL, CR 2–20 μg/mL, CAPE 1–30 μg/mL, and GN 5–50 μg/mL.

#### 4.4.2. Method Validation

The optimized RP-HPLC-DAD method was validated in accordance with the ICH guidelines Q2(R2) and Q14 Guidelines for analytical procedures [33,34] to evaluate selectivity, linearity, sensitivity, precision, accuracy, and robustness.

The selectivity of the method was assessed by comparing representative chromatograms of the mobile phase, standard solutions, propolis samples, a blank sample lacking the target analytes, and a sample spiked with a mixture of standards.

For the linearity assessment, eight calibration points were prepared within the nominal concentration ranges of the polyphenols described in Section 4.4.1. For each calibration level, the mean chromatographic peak area was calculated. The data were evaluated using linear regression to determine the slope and intercept of the calibration curves, as well as the Pearson correlation coefficient (R^2^).

Precision was evaluated by repeated injections of the standard mixture within a single day (repeatability), across several consecutive days (intermediate precision), and through repeated sample preparation, including preparation on a second day using reagents from different production batches. The % RSD was calculated for all measurements.

The limit of detection (LOD) and the limit of quantification (LOQ) for each analyte were determined using the following equations:

LOD = 3.3 × *s*/*slope*

LOQ = 10 × *s*/*slope*

where *s* is the standard residual deviation and the slope of the corresponding calibration curve of each compound.

Accuracy was evaluated by spiking a sample with known concentrations of the analyte mixture at three levels within the working range of the method (LOQ, 100%, and >150% of the nominal polyphenol concentration). Propolis Dub was used as a blank matrix for accuracy testing. Analytical recovery values and % RSD were calculated.

Robustness was examined by assessing the stability of standard and sample solutions in the mobile phase and by evaluating the influence of variations in mobile-phase composition, ambient temperature, injection volume, mobile-phase flow rate, and production batches of reagents and chromatographic columns.

#### 4.4.3. Qualitative and Quantitative HPLC-DAD Analysis

The lyophilized complex obtained as described in Section 4.3 was suspended in HPLC-grade water and subsequently diluted with the mobile phase prior to HPLC analysis. The diluted samples were filtered through 15 mm (i.d.) regenerated cellulose filters with a pore size of 0.45 μm (Agilent Technologies, Waldbronn, Germany).

HPLC analyses were performed using a (U)HPLC Accela system with a DAD detector and a SpectraSYSTEM liquid chromatograph equipped with a UV-6000 detector (Thermo Separation Products, San Jose, CA, USA). Data acquisition and processing were carried out using ChromQuest 5.0 software (Thermo Separation Products, San Jose, CA, USA). Chromatographic separation of polyphenols in the propolis complexes and in vitro fractions was achieved on a ZORBAX Eclipse Plus C-18 column (150 × 4.6 mm, 3.5 μm) coupled with a guard column (14 × 4 mm, 5 μm) (Agilent Technologies, Santa Clara, CA, USA). Isocratic elution was performed using solvent A (water/methanol/formic acid, 97:2:1, *v*/*v*/*v*) and solvent B (methanol) at a 45:55 (*v*/*v*) ratio. The mobile phase was filtered through a 47 mm (i.d.) PTFE filter with a pore size of 1.5 μm (Supelco Analytical, Bellefonte, PA, USA). The flow rate was set to 0.9 mL min^−1^, the column temperature was maintained at 25 °C, and detection was conducted using a DAD detector at 290 nm. The total run time for each analysis was 40 min.

### 4.5. In Vitro Gastrointestinal Digestion

#### 4.5.1. Preparation of Digestion Fluids and Enzyme Solutions

The static in vitro gastrointestinal digestion was performed according to the standardized COST INFOGEST protocol developed by Brodkorb et al. [35], comprising three sequential phases: oral, gastric, and intestinal. Simulated salivary fluid (SSF), gastric fluid (SGF), and intestinal fluid (SIF) were prepared for each digestive phase according to the standardized protocol. Enzyme solutions (α-amylase, pepsin, pancreatin, and bile extract) were freshly prepared and maintained on ice until use. Separate test tubes were employed for each digestion phase to ensure sample homogeneity throughout the procedure. A detailed description of the experimental setup is provided by Perak Junaković et al. [25].

Following completion of the oral, gastric, and intestinal phases, the digested samples were centrifuged at 4472× *g* for 20 min at 4 °C, separating the soluble intestinal fraction (IF-C) from the insoluble residue. The IF-C supernatant was collected and considered the bioaccessible fraction, defined as phenolic compounds released from the food matrix and solubilized under simulated gastrointestinal conditions. The IF-C fractions were used for the quantitative determination of bioaccessible phenolic compounds.

To further assess the potentially absorbable fraction, dialysis was applied to the intestinal supernatant (IF-C). This procedure generated two fractions: the dialyzable fraction (Din), corresponding to compounds capable of diffusing through the semi-permeable dialysis membrane, and the non-dialyzable fraction (Dout), representing compounds retained within the membrane. The Din fraction was used as an indicator of dialyzability, reflecting potential passive intestinal permeability. Importantly, dialysis does not redefine bioaccessibility, but further fractionates the bioaccessible pool into dialyzable and non-dialyzable fractions.

The concentrations of individual standards in the standard mixture and in the HP-β-CD complex during digestion were determined based on the average concentrations of phenolic compounds quantified in the EEP–HP-β-CD complex. Blank samples consisted of water, digestive enzymes, and simulated digestive fluids. Prior to HPLC analysis, aliquots were either diluted or analyzed directly, depending on analyte concentration.

#### 4.5.2. Bioaccessibility and Dialyzability Evaluation

Bioaccessibility and dialyzability were calculated using two complementary in vitro models, namely, a centrifugation-based model, in which bioaccessibility corresponds to the soluble intestinal fraction (IF-C), and a dialysis-based model, in which bioaccessibility corresponds to the total soluble intestinal fraction ((Din + Dout)), while dialyzability represents the fraction of bioaccessible compounds capable of crossing the dialysis membrane.

(1) $$\mathrm{Bioaccessibility}\;(\%)=\frac{content\;of\;compounds\;released\;after\;simulated\;digestion}{content\;of\;compounds\;in\;the\;undigested\;sample}\;\times \;100$$

(2) $$\mathrm{Dialyzability}\;(\%)=\frac{content\;of\;compounds\;passed\;through\;membrane}{content\;of\;compounds\;in\;the\;undigested\;sample}\;\times \;100$$

### 4.6. Statistical Analysis

Samples were analyzed in triplicate, and data are presented as mean ± standard deviation (SD). Statistical analyses were performed using Stata version 13.1 (StataCorp LP, College Station, TX, USA). Data were tested for normality and, as assumptions of normal distribution were not met, nonparametric tests were applied. Differences among digestion phases were evaluated using the Kruskal–Wallis (KW) test. When significant effects were detected (*p* < 0.05), Dunn’s post hoc test was used for pairwise comparisons. To compare the concentration of each polyphenol at individual digestion stages (oral, gastric, intestinal) with its corresponding concentration in the undigested sample (US), Dunn’s post hoc test was applied. The KW test, followed by Dunn’s post hoc test, was used to compare bioaccessibility values of polyphenols between samples depending on the digestion model employed.

## 5. Conclusions

HP-β-CD-based complex systems improved the apparent aqueous solubility, gastrointestinal stability, and intestinal bioaccessibility of major poplar-type propolis polyphenols under the INFOGEST digestion model. The intestinal phase was decisive, with the strongest formulation benefits observed for PC and CAPE, while GN and CA remained more digestion-sensitive and showed matrix-dependent responses. Although bioaccessibility increased markedly, dialyzable fractions remained low, indicating that HP-β-CD primarily enhances luminal availability rather than passive permeability, and that dissociation is likely required prior to absorption. Differences between Valpovo and Zagreb propolis complexes further highlight the influence of source composition on complexation performance and digestive behavior. Overall, HP-β-CD represents a promising, regulatorily accepted platform for more standardized propolis-based nutraceutical and functional formulations, while follow-up studies in cell-based models (e.g., Caco-2) are warranted to better predict intestinal uptake.

## Figures and Tables

**Table 1:** Defined chromatographic conditions for phenolic compound analysis.

Parameter	Description
Column	Agilent ZORBAX Eclipse Plus C-18; 150 × 4.6 mm; 3.5 µm
Column pressure	190 bar
Mobile phase	A:B = 45:55 (*v*/*v*)
Elution mode	isocratic
Column temperature	25 °C
Injection volume	5 µL
Flow rate	0.9 mL min^−1^
Detection wavelength	290 nm

A = water/methanol/formic acid (97:2:1, *v*/*v*/*v*); B = methanol.

**Table 2:** Chromatographic performance parameters of the optimized method.

Parameter	CA	PC	CR	CAPE	GN
Retention time (min)	2.3	20.7	26.5	30.0	33.0
Resolution, Rs	-	50.9	8.2	4.1	3.1
Asymmetry, As	1.6	1.1	1.1	1.1	1.1
Retention factor, k	0.5	10.7	14.1	15.5	17.4
Theoretical plates, N	9490	17,399	17,954	17,111	17,862
Peak area, P	2,985,411	2,898,697	944,844	719,953	1,390,170

CA—caffeic acid; PC—pinocembrin; CR—chrysin; CAPE—caffeic acid phenethyl ester; GN—galangin.

**Table 3:** Validation parameters for the analyzed polyphenols.

Parameter	CA	PC	CR	CAPE	GN
Range (μg·mL^−1^)	2–70	2–70	2–20	1–30	5–50
Standard curve equation	y = 103,076x + 172,530	y = 108,688x − 975.1	y = 80,341x − 101,115	y= 67,299x − 15,473	y = 59,477x − 55,477
R^2^	0.9958	0.9999	0.9999	0.9998	0.9998
LOD (μg·mL^−1^)	1.84	0.23	0.10	0.15	0.31
LOQ (μg·mL^−1^)	5.58	0.69	0.32	0.44	0.93

CA—caffeic acid; PC—pinocembrin; CR—chrysin; CAPE—caffeic acid phenethyl ester; GN—galangin; R^2^—regression coefficient; LOD—limit of detection; LOQ—limit of quantification.

**Table 4:** The results obtained for accuracy evaluation expressed as % Recovery.

Concentration Level	Polyphenol	Nominal (μg mL^−1^)	Mean Found (μg mL^−1^)	Recovery (%)	RSD (%)
Low	CA	6.1	5.4	85.1	1.7
	PC	0.7	1.1	95.9	3.3
	CR	0.7	0.6	81.0	2.2
	CAPE	0.9	0.9	92.4	2.7
	GN	2.1	2.1	101.4	0.7
Medium	CA	27.3	29.6	107.5	2.3
	PC	38.8	41.2	104.9	1.7
	CR	12.8	11.9	92.5	3.6
	CAPE	4.2	4.2	98.0	4.7
	GN	20.6	21.4	104.7	0.7
High	CA	70.9	70.8	99.4	1.2
	PC	68.3	72.4	105.5	1.2
	CR	20.3	19.0	93.4	1.0
	CAPE	31.8	31.8	108.0	0.7
	GN	51.5	42.8	83.0	0.4

RSD—relative standard deviation; CA—caffeic acid, PC—pinocembrin, CR—chrysin, CAPE—caffeic acid phenethyl ester, GN—galangin.

**Table 5:** Aqueous solubility of selected polyphenols in the lyophilized preparations.

Polyphenol	Mass Concentration (μg mL^−1^)		Degree of Enhanced Solubility
	EEP-HP-β-CD	Aqueous Preparation	
CA	248.1 ± 3.9	5224.7 ± 17.5	0.1
PC	1945.2 ± 9.1	340.5 ± 22.2	6
CR	635.4 ± 2.9	35.2 ± 33.7	18
CAPE	211.6 ± 8.0	29.3 ± 15.3	7
GN	993.1 ± 5.0	78.8 ± 20.9	13

Values are presented as mean ± SD. CA—caffeic acid; PC—pinocembrin; CR—chrysin; CAPE—caffeic acid phenethyl ester; GN—galangin.

**Table 6:** Bioaccessibility and dialyzability (%) of phenolic compounds in three samples after simulated gastrointestinal digestion.

Sample	Digestion Outcome	PC	CR	GN	CA	CAPE
Std mix	IF-C/US (%)	77.3 ± 7.2 ^b^	97.2 ± 7.4 ^a^	54.3 ± 4.1 ^b^	28.5 ± 2.5 ^b^	67.4 ± 8.8 ^b^
	Din + Dout/US (%)	80.4 ± 7.8 ^d^	87.0 ± 6.4 ^d^	60.4 ± 3.8 ^d^	31.5 ± 3.2 ^d^	94.2 ± 10.8 ^c^
	Din/US (%)	8.9 ± 0.8	9.8 ± 0.5	0.8 ± 0.1	nd	2.6 ± 0.3
Vlp	IF-C/US (%)	98.2 ± 11.6 ^ab^	89.4 ± 8.2 ^a^	102.3 ± 4.6 ^a^	124.9 ± 25.4 ^a^	112.0 ± 8.3 ^a^
	Din + Dout/US (%)	111.1 ± 9.4 ^d^	121.8 ± 2.4 ^c^	86.4 ± 5.4 ^c^	60.7 ± 7.4 ^c^	99.0 ± 8.4 ^c^
	Din/US (%)	22.9 ± 1.9	26.5 ± 0.4	2.1 ± 0.1	nd	8.8 ± 1.1
Zg	IF-C/US (%)	121.6 ± 13.0 ^a^	77.2 ± 8.9 ^a^	97.4 ± 10.8 ^a^	88.2 ± 15.1 ^a^	104.2 ± 5.4 ^a^
	Din + Dout/US (%)	148.8 ± 11.8 ^c^	97.1 ± 4.9 ^cd^	109.4 ± 5.6 ^c^	85.5 ± 0.1 ^c^	121.6 ± 9.9 ^c^
	Din/US (%)	16.6 ± 1.5	14.8 ± 0.8	1.5 ± 0.1	nd	4.5 ± 0.4

Mean percent values ± standard deviation (SD); *n* = 3. Statistical significance was assessed using the Kruskal–Wallis (KW) nonparametric test followed by Dunn’s post hoc test (*p* < 0.05). Different superscript letters (a, b, c, d) within the same column indicate statistically significant differences (*p* < 0.05) among samples (Std mix, Vlp, Zg) within the same digestion model. IF-C/US and Din + Dout/US represent bioaccessibility (%), while Din/US represents dialyzability (%). nd–not detected or below the limit of quantification. CA—caffeic acid; PC—pinocembrin; CR—chrysin; CAPE—caffeic acid phenethyl ester; GN—galangin; US—undigested sample; IF-C—small-intestinal fraction after centrifugation; Din—small-intestinal fraction inside the semipermeable membrane; Dout—small-intestinal fraction outside the semipermeable membrane.

## Data Availability

The data presented in this study are available on request from the corresponding author.

## Supplementary Materials

The following supporting information can be downloaded at: https://www.mdpi.com/article/10.3390/molecules31040600/s1, Figure S1: Chromatogram showing separation of standards of caffeic acid (CA), pinocembrin (PC), chrysin (CR), caffeic acid phenethyl ether (CAPE), and galangin (GN) under the optimized HPLC method (1: CA, 2: PC, 3: CR, 4: CAPE, 5: GN); Figure S2: Chromatogram showing separation of standard polyphenol mixture: caffeic acid (CA), pinocembrin (PC), chrysin (CR), caffeic acid phenethyl ether (CAPE), and galangin (GN) under the minor modifications of mobile phase (1: CA, 2: PC, 3: CR, 4: CAPE, 5: GN). Figure S3: Chromatogram of the propolis aqueous preparation without HP-β-CD; Figure S4: Chromatogram showing polyphenols CA, PC, CR, CAPE, and GN in the propolis complex in the undigested sample (black) and the dialyzable fraction Din (violet) (1: CA, 2: PC, 3: CR, 4: CAPE, 5: GN); Table S1: Precision was based on nominal concentration measurements of the polyphenols in the propolis complex.
