# Supplementary material for: 2-Hydroxypropyl-β-Cyclodextrin-Based Complexes Improve Polyphenol Solubility and Bioaccessibility: Evaluation by Validated HPLC–DAD Method
Source: Molecules. 2026 Feb 9;31(4):600. doi: 10.3390/molecules31040600 (PMC12942958; doi:10.3390/molecules31040600)
Supplement: Supplementary file 1 [file molecules-31-00600-s001.zip › Suplementary files_table.pdf]

Table S1. Precision was based on nominal concentration measurements of the polyphenols in the propolis complex

**Phenolic compound**	**Precision (Mean %RSD)**	
	**Intraday**	**Interday**
**CA**	**6.7**	**3.8**
**PC**	**5.4**	**4.4**
**CR**	**6.5**	**8.5**
**CAPE**	**8.0**	**1.9**
**GN**	**6.5**	**5.9**
