# Supplementary material for: 2-Hydroxypropyl-β-Cyclodextrin-Based Complexes Improve Polyphenol Solubility and Bioaccessibility: Evaluation by Validated HPLC–DAD Method
Source: Molecules. 2026 Feb 9;31(4):600. doi: 10.3390/molecules31040600 (PMC12942958; doi:10.3390/molecules31040600)
Supplement: Supplementary file 1 [file molecules-31-00600-s001.zip › Supplementalry file_figures.pdf]

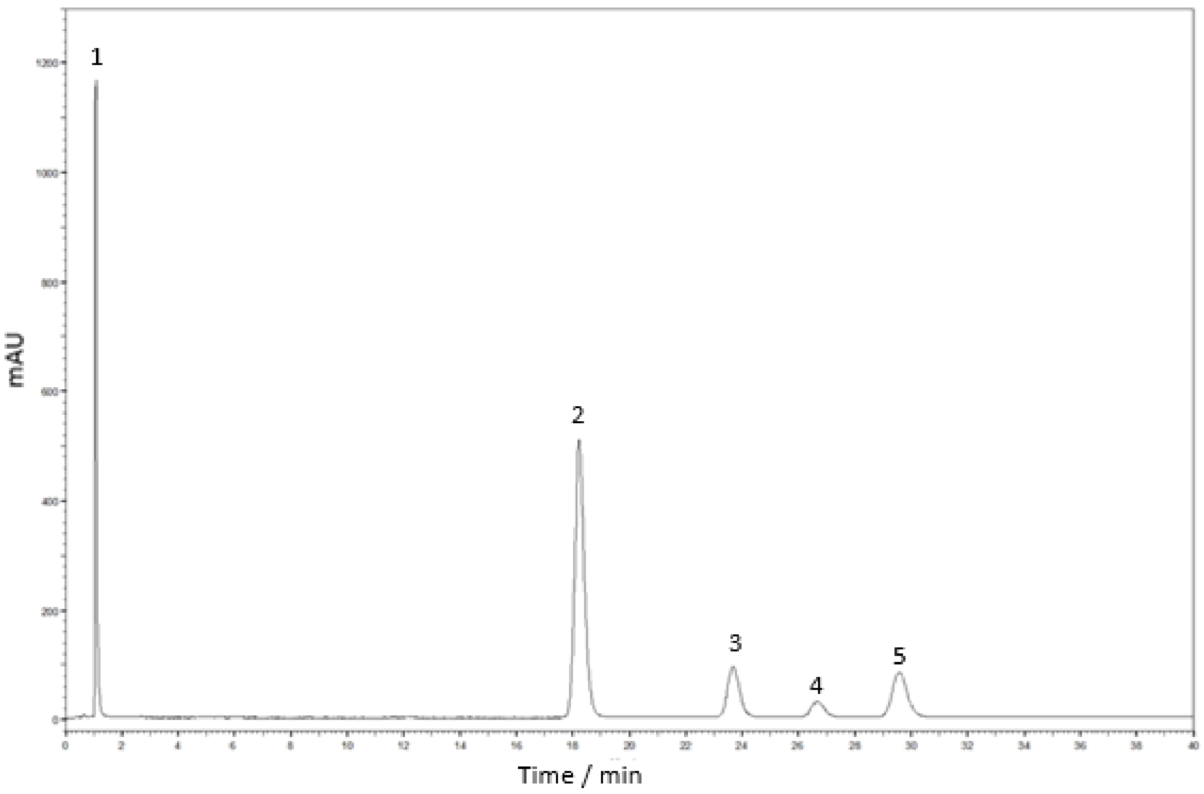

Figure S1. Chromatogram showing separation of standards of caffeic acid (CA), pinocembrin (PC), chrysin (CR), caffeic acid phenethyl ether (CAPE), and galangin (GN) under the optimized HPLC method (1: CA, 2: PC, 3: CR, 4: CAPE, 5: GN)

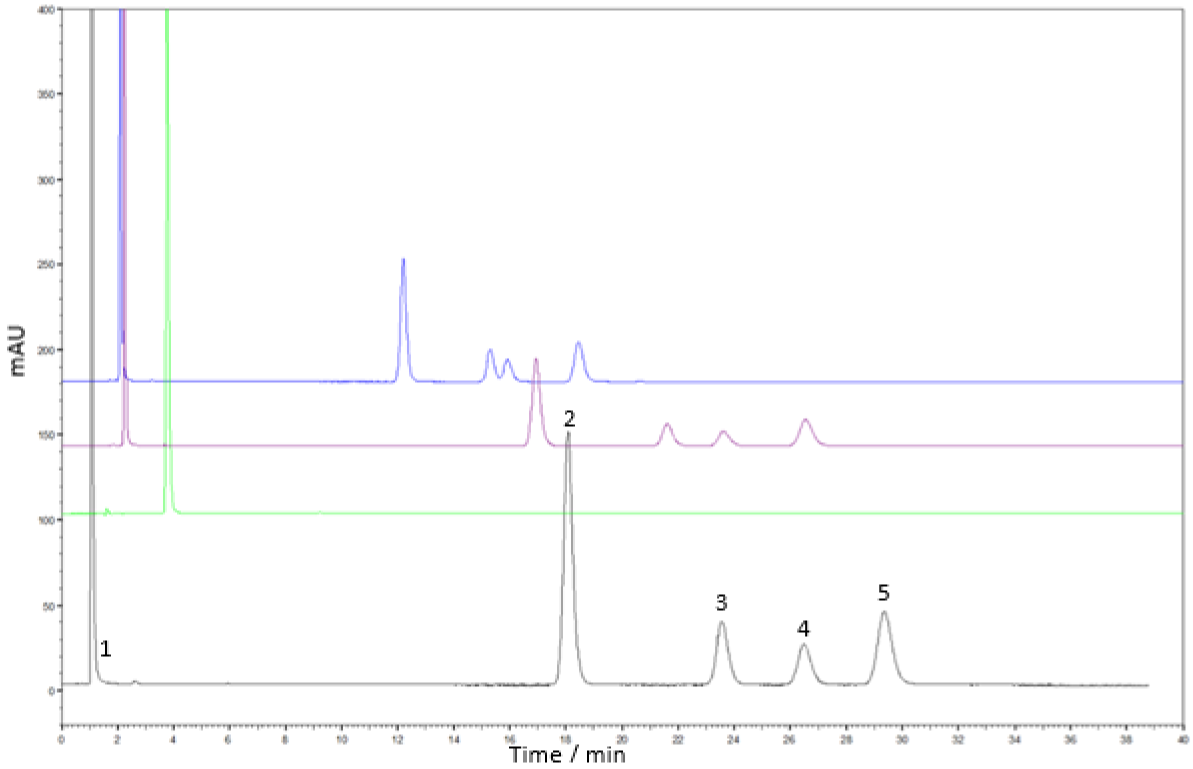

Figure S2. Chromatogram showing separation of standard polyphenol mixture: caffeic acid (CA), pinocembrin (PC), chrysin (CR), caffeic acid phenethyl ether (CAPE), and galangin (GN) under the minor modifications of mobile phase (1: CA, 2: PC, 3: CR, 4: CAPE, 5: GN).

(➡) A : B = 45 : 55; (➡) A : B = 40 : 60; (➡) A : B = 42,5 : 57,5; (➡) A : B = 60 : 40

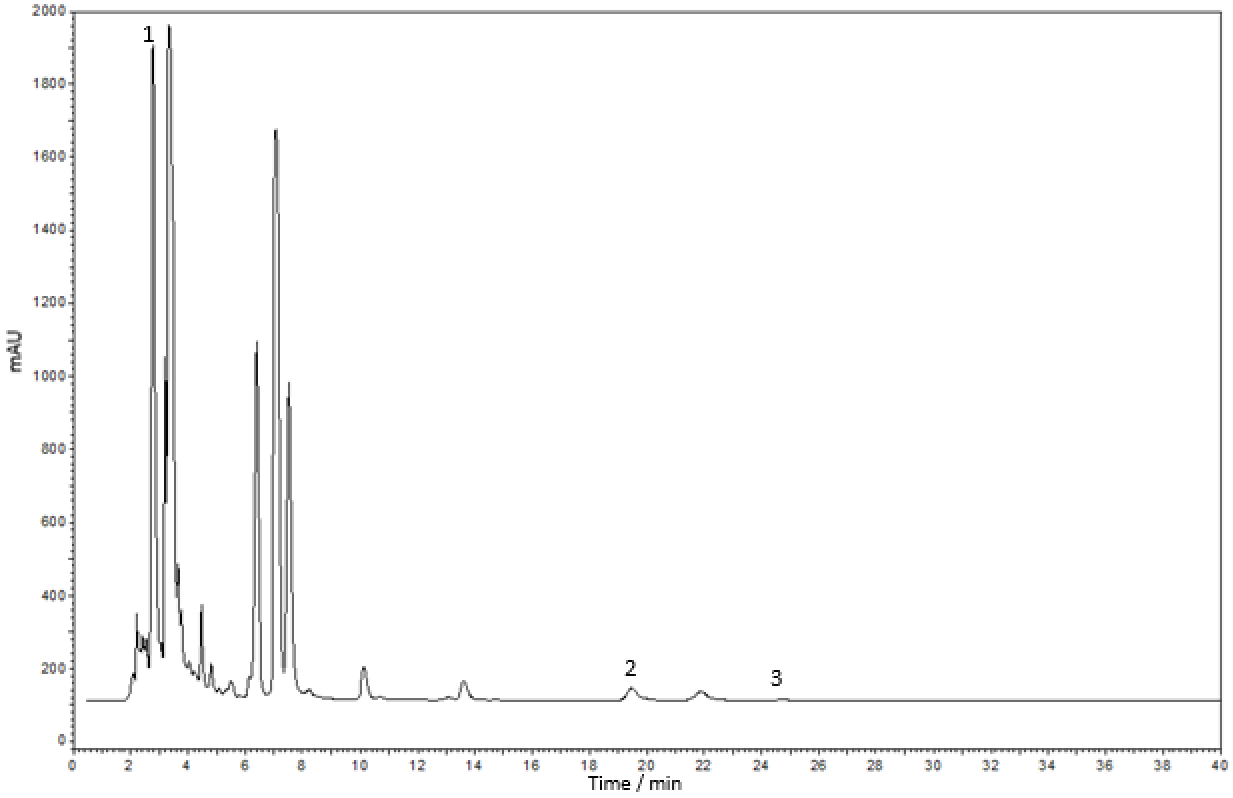

Figure S3. Chromatogram of the propolis aqueous preparation without HP-β-CD (1: CA, 2: PC, 3: CR)

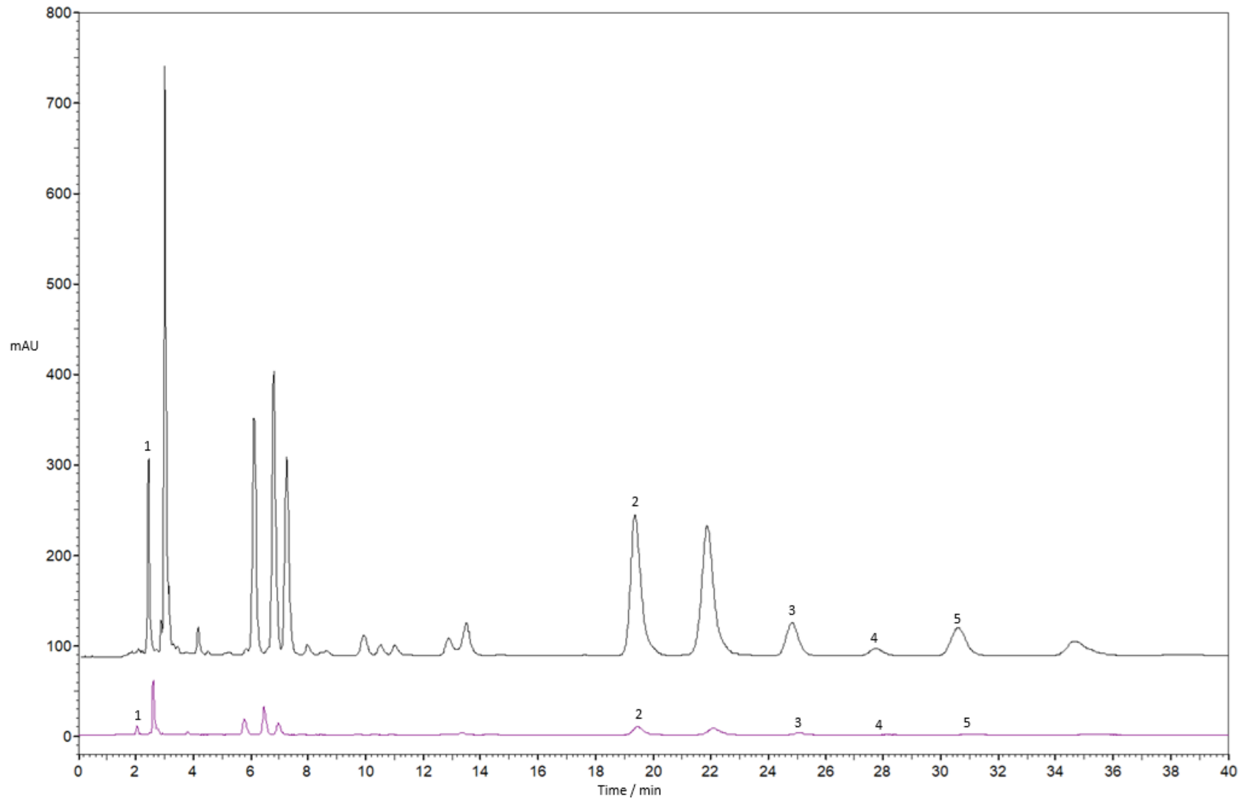

Figure S4. Chromatogram showing polyphenols CA, PC, CR, CAPE, and GN in the propolis complex in the undigested sample (black) and the dialyzable fraction Din (violet) (1: CA, 2: PC, 3: CR, 4: CAPE, 5: GN)
